# Rapid Emergence of Multidrug-Resistance among Gram Negative Isolates at a Tertiary Pediatric and Maternity Hospital in Ulaanbaatar, Mongolia

**DOI:** 10.5195/cajgh.2020.371

**Published:** 2020-04-27

**Authors:** Susanna Felsenstein, Sarantsetseg Bira, Narangerel Altanmircheg, Enkhtur Shonkhuuz, Ariuntuya Ochirpurev, David Warburton

**Affiliations:** 1 Department of Paediatric Infectious Diseases and Immunology, Liverpool, United Kingdom; 2 Central Laboratory Department, National Center for Maternal and Child Health, Ulaanbaatar, Mongolia; 3 Critical Care Medicine, National Center for Maternal and Child Health, Ulaanbaatar, Mongolia; 4 Health emergencies and food safety, Office of the WHO Representative in Mongolia, Ulaanbaatar, Mongolia; 5 Keck School of Medicine, University of Southern California, USA; 6 Ostrow School of Dentistry, University of Southern California, USA

**Keywords:** Gram Negative Bacterial Infections, Beta Lactamases, Drug Resistance, Multiple, Intensive Care Units, Mongolia, Stewardship

## Abstract

**Introduction:**

Information on microbiological and susceptibility profiles of clinical isolates in Mongolia is scarce, hampering infection control and clinical care.

**Methods:**

Species and resistance profiles of 6334 clinical gram negative isolates, collected at Mongolia's National Center for Maternal and Child Health between 2014 and 2017 were analyzed.

**Results:**

Annual proportion of multidrug-resistance among *E. coli* and *Enterobacter* isolates increased from 2.8% to 16.6% and 3.5% to 22.6% respectively; *Klebsiella* isolates exhibiting susceptibilities suggestive of extended spectrum beta-lactamase (ESBL) production from 73% to 94%. By 2017, 60.6% of *Klebsiella* isolates were multidrug-resistant, most originated from intensive care wards. Enterobacteriaceae exhibiting susceptibility patterns suggestive of ESBL production and multidrug-resistant organisms were common and their incidence increased rapidly.

**Conclusion:**

These findings will serve to build strategies to strengthen microbiological surveillance, diagnostics and infection control; and to develop empiric therapy and stewardship recommendations for Mongolia's largest Children's and Maternity hospital.

## Introduction

Antimicrobial resistance (AMR) in Asia increases at concerning rates^1^^–^^6^. The World Health Organization (WHO) projects an attributable cost of 700.000 lives annually and 1.35 trillion USD in countries of the Western Pacific Region (WPRO) alone for the next decade^7^.

Numerous, rapidly evolving and readily transmissible mechanisms of antimicrobial resistance in gram negatives, particularly the rise in carbapenem resistant enterobacteriaceae over the last two decades is of grave concern, and the cause of significant morbidity and mortality^8^^,^^9^. Data on AMR in Mongolia is scarce, and the country is not part of transnational surveillance networks^6^^,^^10^. A surveillance system at urban and rural level training health care providers in infectious diseases management and control is established but implementation is challenging^10^^,^^11^. However, the Mongolian National Statistical Office and the United Nations International Children's Emergency Fund (UNICEF) report a four-fold reduction in infant and under 5's mortality over the last two decades, mainly by control of respiratory and vaccine-preventable diseases^12^^,^^13^, illustrating the major strides made in improving child and maternal health.

Mongolia features one of the lowest population densities worldwide. Approximately half of the 3 million inhabitants reside in the capital Ulaanbaatar, the remainder in provincial capitals, many of which follow a traditional nomadic lifestyle. The country's economy is growing rapidly, mainly as a result of an expanding mining industry^12^. Despite rapid modernization, especially in Ulaanbaatar, many parts of the country are remote and not readily accessible. Hence, challenges in combating the spread of AMR are complex^10^^,^^14^. Health care provision and access, development of diagnostic facilities, staff education, antimicrobial surveillance, auditing of prescription practices and enforcement of drug regulation remain a challenge.

The National Center for Maternal and Child Health (NCMCH) is the largest government-run pediatric and maternity hospital and only tertiary referral center. The pediatric hospital has 270 medical and 150 surgical beds accommodating 19 subspecialties and treats approximately 40.000 inpatients and over 175.000 outpatients each year. The adjacent 250-bedded maternity hospital provides gynecological and obstetric care, and manages approximately 12.000 deliveries per annum^15^.

The aim of this study is to provide pathogen and susceptibility data for gram negative organisms (GNOs) among in- and outpatients of different ages in Ulaanbaatar; allowing the development of evidence-based empirical antimicrobial treatment and infection control policies.

## Methods

### Data collection

All gram negative isolates of cultured specimens submitted to the microbiology laboratory at NCMCH between 01/2014 and 08/2017 were included. Data was collected via the WHONET database (version 5), retrospectively (2014-2016) and prospectively (2017), including demographic data, specimen type, in- /outpatient status, hospital ward, species identification and antimicrobial susceptibility testing (AST) profile, and adequacy of microbiological work-up. The study was approved by the NCMCH ethics committee (NCMCH-AS2014).

### Susceptibility testing

At times, test panels differ from the Clinical and Laboratory Standards Institute (CLSI) and the European Committee on Antimicrobial Susceptibility Testing (EUCAST) guidelines due to limitations of AST available. Additions or omissions to standard test panels are specified in the text. Organisms identified by means other than culture and stool samples are processed at another facility and were excluded. Molecular detection of organisms and/or determination of susceptibility status was not available during the study period.

Of identical organisms isolated from a patient within 30 days, only the first isolate was included. Only species with a minimum of 30 isolates per year, per specimen category and antimicrobial agent tested, were used for guiding empirical antimicrobial recommendations. Susceptibility is interpreted as per CLSI 2016 and CESAR (Central Asian and Eastern European Surveillance of Antimicrobial Resistance) guidelines^6^^,^^16^. Resistant and intermediately resistant isolates are reported as one category (%R/I). AST to antimicrobials not recommended for treatment of an organism are excluded except for service utilization analysis. Confirmation of phenotypical presence of extended spectrum betal lactamases (ESBL) by Double Disk or minimal inhibitory concentration (MIC) and confirmation of AmpC status was not available. ESBL status attribution relied on disk diffusion interpreted as per CLSI, CDC and CESAR recommendations^6^^,^^14^^–^^19^. Species isolates assumed to be AmpC-producers were excluded from ESBL analysis.

Carbapenem susceptibility testing became available in July 2016. Classification of carbapenem susceptibility used the most conservative estimate: if tested for one carbapenem only or if the test result was concordant, the report was analyzed as the final result. Discordant carbapenem susceptibilities, with resistant or intermediate susceptibility for at least one, were reported as carbapenem resistant (CR)^6^^,^^19^. Resistance to antibiotic classes was reported by combining the results of antibiotics representative for a class and the outcome based on the most resistant result. Multidrug-resistance (MDR) was defined as resistance to three or more antimicrobial classes in isolates with a valid result for at least three, calculated as resistance or intermediate resistance to at least one antibiotic in each of class^19^, and documented by number of antimicrobial classes to which resistance was identified.

### Statistical analysis

Quantitative variables were reported as absolute numbers and percentages. For continuous variables comparisons between groups to test equality were performed using the t-test when appropriate, or Mann-Whitney test when skewed. Tests of association between categorical variables were based on Chi-square and Fisher-Exact Tests. All P values reported are two-sided and considered statistically significant if < .05. Statistical computations were performed using SPSS 22.0 (SPSS Inc. Chicago, Illinois).

## Results

Epidemiology and origin of isolates: In total, 6334 (53.3%) GNOs were included. *E. coli* and *Enterobacter spp.* were most common, accounting for 90% of isolates (Table 1).

**Table 1.** Species identification of gram negative isolates.

Organism	2014	2015	2016	2017	Total
***Escherichia coli***	1220 (67.3%)	1379 (57.1%)	602 (40.4%)	78 (12.6%)	3279 (51.8%)
***Enterobacter***	510 (28.1%)	873 (36.2%)	648 (43.5%)	417 (67.5%)	2450 (38.6%)
*E. cloacae*	327	859	472	78	1736
*Enterobacter spp. (unidentified)*	172	2	163	335	672
*E. aerogenes*	10	7	7	4	28
*E. sakazakii*	0	2	6	0	8
*Pantoea spp.*	0	3	0	0	3
*P. agglomerans*	1	0	0	0	1
***Klebsiella***	26 (1.4%)	70 (2.9%)	119 (8.0%)	67 (10.8%)	282 (4.5%)
*Klebsiella spp. (unidentified)*	5	1	65	62	133
*K pneumoniae*	20	53	13	0	86
*K oxytoca*	1	16	41	5	63
***non-aer. Pseudomonas spp., Chryseobacterium, Flavimonas***	5 (0.3%)	10 (0.4%)	62 (4.2%)	38 (6.1%)	115 (1.8%)
***Pseudomonas aeruginosa***	17 (0.9%)	38 (1.6%)	11 (0.7%)	0	66 (1.0%)
***Proteus mirabilis***	34 (1.9%)	19 (0.8%)	24 (1.6%)	12 (1.9%)	89 (1.4%)
***Raoultella spp***	2 (0.1%)	12 (0.5%)	9 (0.6%)	1 (0.2%)	24 (0.4%)
*R. ornitholytica*	1	4	7	1	13
*R. terrigenica*	0	8	2	0	10
*R. planticola*	1	0	0	0	1
***Serratia odifera***	0	8 (0.3%)	0	0	8 (0.1%)
***Serratia marescens***	0	2 (0.1%)	1	2 (0.3%)	5 (0.1%)
***A cinetobacter baumannii***	0	0	6 (0.4%)	0	6 (0.1%)
***Eliz abethkingia meningoseptica***	0	0	4 (0.3%)	1	5
***Burkholderia cepacia***	0	0	1	1	2
***Kluyvera***	0	0	1	0	1
***Chromobacterium violaceum***	0	1	0	0	1
***Salmonella***	0	1	0	0	1
***Pasteurdla spp***	0	0	1	0	1
***Moraxella***	0	0	0	1	1
**Total**	1814	2413	1489	618	6334

**Figure 1.**
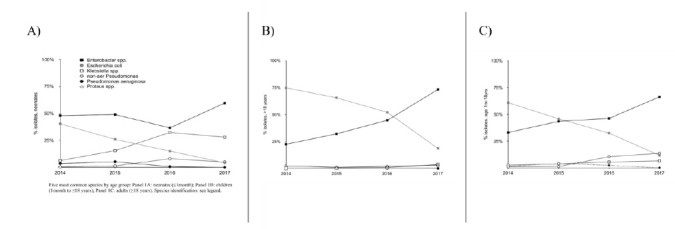
Five most common species by age group: Panel 1A: neonates (≤1month); Panel 1B: children (lmonth to ≤18 years); Panel 1C: adults (≥18 years).

**Figure 2.**
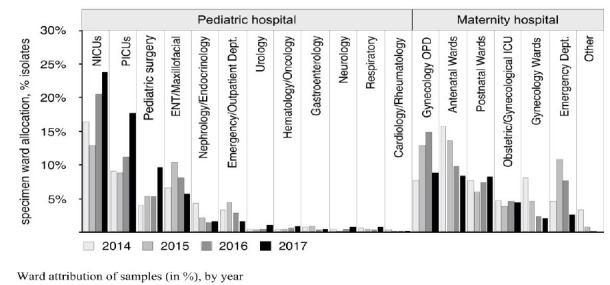
Ward attribution of samples (in %), by year

Neonatal samples were dominated by wound (24.9%) and blood stream isolates (37.1%) (BSI); pediatric samples by wound (28.8%) and urinary (42.3%), isolates from adults by genital (63.5%) and urinary (30.8%) samples. Predominant GNOs differed considerably depending on age group (Figure 1). Sample numbers submitted from neonatal (NICU) and pediatric intensive care (PICU) units increased over time, accounting for 45.8% of all isolates by 2017 (Figure 2).

*Enterobacter* spp.: Frequency of *Enterobacter spp.* isolates increased over time. Most were inpatient isolates (n=2051, 84%), almost half (n=1078, 44%) from neonates and children. The annual increase of *Enterobacter spp.* among neonatal BSIs was significant (P<0.001), contributing to a third of gram negative BSIs (42/140; 30%) in 2017.

Whilst in 2014/15 *Enterobacter* susceptibility to quinolones and aminoglycosides was reliable, resistance increased sharply from 2016 (Supplemental Figure 1A), especially among inpatients and BSIs. From 2017, virtually all *Enterobacter spp.* blood isolates were aminoglycoside resistant (46/47, 97.9%). In the first half of 2017 alone, 57/277 (20.6%) of *Enterobacter* isolates tested were carbapenem resistant (CR), particularly blood (17/47; 36.2%) and urinary isolates (25/87; 28.7%). CR *Enterobacter* isolates originated mainly from NICU (47/93; 50.5%) and PICU (30/93; 32.3%).

**Figure 3.**
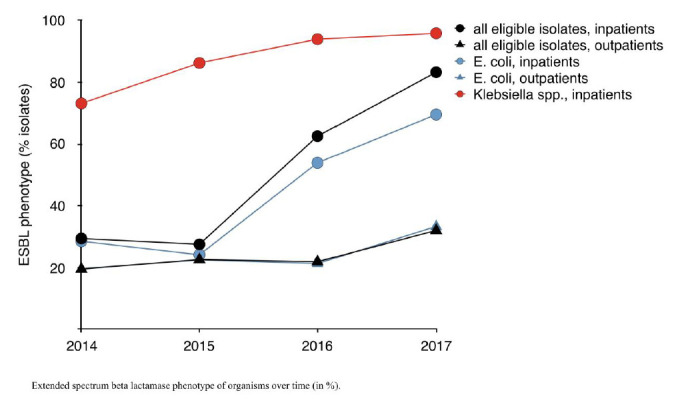
Extended spectrum beta lactamase phenotype of organisms over time (in %).

**Figure 4.**
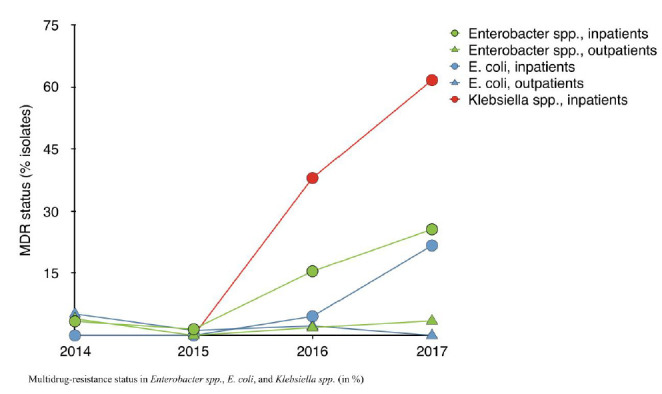
Multidrug-resistance status in Enterobacter spp., E. coli, and Klebsiella spp. (in %)

*E. coli:* Among *E. coli,* adult samples accounted for 75.1% (n=2459/3274), predominantly from urine (n=1207, 36.8%) or genital tract (n=1498, 45.8%). The number of *E. coli* isolates fulfilling criteria for ESBL confirmatory testing doubled over the study period from 27.0% to 60.3%; attributable to inpatient and NICU isolates (Figure 3). Aminoglycoside susceptibility remained stable at 75% whilst quinolone susceptibility decreased, one third of isolates were no longer susceptible by 2017 (Supplemental Figure 1B). Only 5.3% (172/3246) of *E. coli* isolates underwent Carbapenem susceptibility testing. The proportion of CR isolates increased from 12.8% (2016) to 28.2% (2017), almost all were inpatient samples (29/32), 72% (n=23/32) from ICUs (Supplemental Figure 1B). MDR increased between 2016 and 2017 among *E. coli* from 2.8% to 16.6% (Figure 4).

*Klebsiella* spp.: Almost all *Klebsiella* isolates, 98.2% (277/282) originated from inpatients, most (208/282, 73%) from NICU, 66.3% of which were BSIs (138/208); the remainder wound (9.2%) and urine isolates (6.7%). In nine instances, *Klebsiella* was isolated from neonatal CSF. The majority showed susceptibility patterns suggestive of ESBL production, most frequently among isolates from neonates (adults: 64%, 16/25; children: 83.1%, 49/59; neonates: 94.4%, 187/198), but increasing across all age groups from 73% in 2014 to 94% in 2017. Most were gentamicin resistant by 2017 (53/64, 83%), as opposed to only 25% (6/24) in 2014. Again, this was most pronounced among neonates, where aminoglycoside resistance increased from 26% (n=23; 2015) to 95.7% (n=46; 2017). Quinolone resistance increased from 5.5% (3/55; 2015) to 60.6% (40/66; 2017) (Supplemental Figure 1C). Only 135/282 (48%) underwent carbapenem susceptibility testing, 24.7% (19/77; 2016) and 28.1% (16/57; 2017) were identified as CR and originated mainly from NICU, where CR rates increased from 13.8% (8/58, 2016) to 32.5% (14/43, 2017).

Remaining GNOs, including *Pseudomonas* spp., *Raoultella* spp., *Serratia* spp., *Acinetobacter* spp.: Documentation of specimen origin was only available for 42/66 (63.6%) of *P. aeruginosa* isolates, urines and wound swabs predominated. Reduced susceptibility to ceftazidime (13/54; 24%), gentamicin (12/62, 19.3%) and quinolones (8/55; 14.5%) occurred, however isolate numbers with susceptibility data available for all antimicrobials of interest were lower than 30 per year. All multidrug resistant isolates originated from NICU (n=3) and PICU (n=2), carbapenem susceptibility was not documented.

Samples yielding isolates of the non-aeruginosa *Pseudomonas spp.* group increased significantly (p<0.001) with PICU (n=42) and NICU (n=42) contributing most isolates. The majority did not undergo susceptibility testing.

*Raoultella spp.* were mainly isolated in 2015/16, when 16/24 isolates, mainly *R. terrigenica* of identical susceptibility pattern, were isolated from specimens of multiple wards. Most *Serratia* isolates were identified in 2015 (10/13), mainly on neonatal wound swabs and BSIs, and genital samples from maternity wards. Quinolone susceptibility was universal (13/13; 100%), a quarter were aminoglycoside resistant. Infections with *Chryseomonas luteola* occurred exclusively in November/December 2015; isolated from urine (n=3), pleural (n=2) and peritoneal (n=2) fluid; and confined to NICU (n=5) and the pediatric nephrology ward (n=4).

*Acinetobacter baumannii* was isolated in six patients: four from neonatal BSIs and one from neonatal CSF and a joint aspirate of a young child - all in November 2016. All were quinolone, aminoglycoside and carbapenem susceptible.

ESBL production and Multidrug resistance across species over time: Rates of ESBL phenotype diverged when comparing in- and outpatients in ESBL producers (Figure 3). In 2014, 29% of in- and 19.5% of outpatient isolates would have required confirmatory testing, by 2017, this applied to 83% respectively 32% (*P*<0.001).

MDR increased significantly more among inpatients, too (*P*<0.001, Figure 4). In earlier years, only half of *Klebsiella* and *Enterobacter* isolates could be included, as the choice of antimicrobials tested for were not agents recommended for treatment, or susceptibilities for too few antimicrobials were documented, however adequate microbiological work up was achieved for almost all isolates by 2017. MDR among *E. coli* tested increased from 2.8% in 2014 to 17.2% by 2017; among *Enterobacter* from 3.5% to 22.6%. The increase in MDR was most pronounced among *Klebsiella spp.* where no multidrug-resistance was identified in 2014/15, affected a third in 2016 and doubled within a year to 60.6% of isolates by 2017.

## Discussion

This data represents the largest published dataset on pathogen and resistance profile of clinical GNOs from Mongolian patients. Mongolia's health care system has evolved to apply novel therapeutic options, that are associated with an increasing use of antimicrobials for nosocomial infections in intensive care settings and hence accompanied by new challenges in governance and stewardship.

With ESBL confirmation testing indicated for the majority of inpatient isolates, this study identified an urgent need for implementation of improved microbiological diagnostics enabling accurate identification of drug-resistant GNOs; and steps are being taken to address it^15^. Confirmatory testing is being introduced as a result of the here presented data, which illustrates an improvement in the detection of MDR and ESBL status since 2017 as a result of these efforts. Staff training supported by online resources and international collaborations is being undertaken^9^. More standardized susceptibility testing is being done, to first line antimicrobials initially, followed by second line testing where indicated, and ESBL and Carbapenemase testing is done where indicated. All *Klebsiella* isolates will undergo AST for quinolones and aminoglycosides.

It must be acknowledged that the present study has important limitations. First, a more detailed molecular and genotypic characterization of the organisms would have been of interest, at both local level - impacting infection control and treatment options - and on a transnational level, putting Mongolian isolates into context within Asia. Financial restraints and the need for prioritization within the healthcare sector have hampered molecular diagnostics at NCMCH to date, and diagnostic possibilities for AST remains limited. We found that the microbiological work up was not always in keeping with recommendations made by professional agencies ^6^^,^^16^^–^^19^ Especially the lack of ESBL confirmation posed difficulties, limiting treatment recommendations that could be inferred.

The results of the available testing presented here however are concerning and serve as an opportunity to put AMR in Mongolia at the forefront of public health policies in the months and years to come. It is of utmost importance that data is made accessible to clinicians and to serve as an indicator for stakeholders that AMR poses a clinical risk at NCMCH, and in the Mongolian capital at large.

Only a minority of *E. coli* and *Enterobacter spp.* isolates underwent Carbapenem susceptibility testing, the data may not reflect true susceptibility rates. A sizeable proportion of isolates underwent testing for less than three antimicrobials, disqualifying them from MDR analysis, particularly impacting *Enterobacter* and *Klebsiella spp.* isolates.

Whilst the representation of phenotypical ESBL status may overestimate the true rate of ESBL producers by 5-30%^20^, it is concerning to see such rapid increase in organisms of ESBL phenotype. Data on the resistance among uropathogenic *E. coli* in Mongolia from 2016^21^ supports our findings of a high burden of ESBL and MDR, identifying resistance to beta-lactams in over 80%. Susceptibility to nitrofurantoin and imipenem was relatively preserved, but 93.9% of *E. coli* isolates were multidrug resistant. While in this cohort, MDR rates among *E. coli* were lower, proportion of CR isolates was much higher. Testing to more antimicrobial classes, including Co-trimoxazole and Nitrofurantoin^21^, may have contributed to this discrepancy. In addition, we excluded 643 *E coli* isolates from MDR analysis, as they underwent testing for fewer than three antimicrobials. However, our data encompasses a larger sample; and results did not change upon subanalysis of urine isolates (n=1201): 13.4% were multidrug resistant, relying on gentamicin, quinolone, beta-lactams and, since 2016, carbapenem testing.

Kao et al^22^ report findings closely resembling our study: in 104 isolates from NCMCH in 2013, 18% of *E. coli* isolates were ESBL producers, mediated by TEM1 and associated with fluoroquinolone resistance. Our data shows rising rates the following year, identifying 27% of *E. coli* inpatient- and 19% of outpatient isolates as ESBL producers. The diagnostics available at NCMCH may influence ESBL rates, underlining the need for rapid introduction of ESBL confirmatory testing, not only to ensure accurate surveillance but also to avoid unnecessary use of carbapenems. Given that ESBL rates in our cohort doubled between 2014 and 2017 from 27% to 60%, the difference between the two studies may reflect the true increase in ESBL rates over time.

In summary, the here presented data is in keeping with studies reporting susceptibilities that included the confirmation of ESBL. As no significant changes to the diagnostic work up occurred during the study period it has to be assumed that the significant increase in multidrug resistance, ESBL production and Carbapenem resistance may well reflect an approximation of the true development of AMR, which was mainly driven by inpatient isolates. This information may represent a chance to effectively impact spread of MDR and ESBL positive GNOs in Mongolia by intensifying infection control in health care facilities.

Proportion of ESBL phenotype and carbapenem resistance was extraordinarily high among nosocomially acquired *Klebsiella* BSIs on NICU, steeply rising since 2016. CR affected one third of *Klebsiella* isolates on NICUs by 2017. In keeping with reports from ICUs globally, Carbapenemase-positive Klebsiella has been included in the WHO global priority pathogen list^23^.

Given the level of concern due to these results, a detailed assessment of clinical and infection control practices on NICUs was performed. NICU staff were aware of the high prevalence of *Klebsiella,* and despite empiric treatment of neonatal BSIs with imipenem, neonatal demise due to *Klebsiella* BSIs whilst on imipenem therapy was reportedly common, though no mortality data was available. Neonatal PICC lines were not routinely used, and staff did not feel competent in sterile line insertion. Nursing staff were unfamiliar with line care, hub sterilization before flushing and connecting, fixation of lines etc. Newborns requiring antibiotic treatment or total parenteral nutrition underwent frequent peripheral re-cannulations, performed routinely every three days.

In the interim, guidelines addressing infection control procedures, central venous access insertion and line care have been provided to staff. Sterile line insertion was supervised and training provided. The introduction of medium-term, silver or heparin coated long lines for neonates has been discussed^24^. Ongoing data collection on central line access related BSIs on NICU including associated morbidity and mortality following these interventions will inform future clinical practice.

Our study emphasizes the preeminent role BSIs have played in the overall increase of MDR at NCMCH, especially affecting intensive care units. With a use of ICU beds in Mongolia equivalent to that of Western European or North American countries at 11.7 per 1.000.000 inhabitants^25^, the intensive care environment appears to be a major contributor to the emergence of AMR. CR and MDR in ICUs is well recognized to be a pressing issue in other countries in Central Asia, such as Kazakhstan^26^.

The largest published record of clinical GNOs in Mongolia to date emphasizes the importance of consequent surveillance and raises attention to multidrug-resistance in Central Asia, and highlights the intensive care environment as important source of AMR emergence^27^. The data presented here will form part of the National Action Plans including surveillance systems nested under the multi-sectoral framework and the One Health Approach of the WHO for Mongolia^28^^,^^29^. International collaboration, financial reprioritization and educational support for health care professionals will be required in order to assist measures combating the spread of AMR in Mongolia.
